# The Impact of Real‐Time Data Analytics on Infection Prevention and Control Practices in Ophthalmology: A Nursing Perspective on Patient Outcomes and Cost‐Effectiveness

**DOI:** 10.1111/nhs.70327

**Published:** 2026-04-05

**Authors:** Zhenhui Chen, Shengcheng Wu, Yi Zhou, Chun Tang

**Affiliations:** ^1^ Nursing Department, Shanghai Eye Diseases Prevention & Treatment Center/Shanghai Eye Hospital, School of Medicine Tongji University Shanghai China; ^2^ Ophthalmology Department, Shanghai Eye Diseases Prevention & Treatment Center/Shanghai Eye Hospital, School of Medicine Tongji University Shanghai China

**Keywords:** cost‐effectiveness analysis, medical costs, nursing, ophthalmic infection prevention and control, real‐time data analysis

## Abstract

Ophthalmic surgeries pose infection risks. Traditional control methods rely on manual monitoring, creating a need for more precise, data‐driven nursing models. This study evaluated the effectiveness of real‐time data analysis in improving ophthalmic infection prevention and control outcomes. This study was designed as a single‐center retrospective study. We analyzed 213 patients (2022–2024). The conventional group (*n* = 105) received conventional care, while the real‐time data analytics group (*n* = 108) received real‐time data‐driven infection prevention and control. Measured outcomes included infection rates, complications, visual acuity, quality of life, visual function, pain scores, costs, and cost‐effectiveness. The real‐time data analytics group showed significantly better outcomes: lower infection rates (*p* = 0.032), better visual acuity (*p* = 0.042), and less pain (*p* = 0.024). They also had higher quality of life (*p* = 0.036) and visual function scores (*p* = 0.032). Direct medical costs and nursing costs were significantly lower (*p* < 0.001), with the ICER was −11656.22 yuan/QALY, indicating a dominant economic result. Real‐time data analysis enables dynamic risk monitoring and precise interventions in ophthalmic nursing. This approach reduces infections, improves visual outcomes, lowers costs, and enhances cost‐effectiveness, supporting standardized quality improvement in infection prevention and control.

## Key Points

- Superior outcomes: Compared with conventional care, nursing interventions based on real‐time data analytics are more effective in reducing infection rates, improving visual recovery, and enhancing the patient experience.
- High economic feasibility: This model has the potential to improve healthcare quality while simultaneously reducing the costs associated with individual infections and nursing labor. It demonstrates a more favorable incremental cost‐effectiveness ratio (ICER), indicating strong cost‐effectiveness potential.
- Clear mechanism of action: The positive effects stem from dynamic data monitoring, precise risk identification, and targeted optimization of protocols, thereby providing a reference pathway for standardizing infection prevention and control (IPC) in ophthalmology.

## 1 Introduction

Visual health is directly related to an individual's quality of life, independent living ability, and social productivity. Among these services, invasive procedures such as phacoemulsification for cataracts, trabeculectomy for glaucoma, and vitrectomy account for 68.3%. Such procedures require direct contact with intraocular tissues or damage to the ocular surface barrier (Hossain et al. 2023; Pucchio et al. 2023), making ocular infection a core risk that cannot be ignored in ophthalmic diagnosis and treatment. Even a mild infection may, due to the fragility and functional particularity of ocular tissues, lead to severe complications such as corneal ulcers and endophthalmitis, ultimately resulting in permanent visual impairment or even enucleation. This not only brings double physical and psychological trauma to patients but also imposes a heavy burden on families and society (Durand et al. 2023; Valek et al. 2023).

At present, ophthalmic infection prevention and control (IPC) in most hospitals in China still relies on the traditional model of “manual monitoring + empirical intervention”. Centered on the subjective experience of nursing staff, this model conducts prevention and control work through regular inspections, manual recording of infection‐related data, and routine postoperative use of antibiotics (Hill et al. 2024). Under the traditional nursing model, nursing staff's monitoring of infection risks relies on scheduled and fixed‐point manual observation. This intermittent monitoring model cannot capture real‐time dynamic changes. Due to the inability to integrate patients' multi‐dimensional data in real time, infection intervention plans under the traditional nursing model are mostly standardized templates (Kang and Tanna 2021). The traditional plan cannot meet such individualized needs. More seriously, the blind use of broad‐spectrum antibiotics may also lead to increased bacterial resistance. In addition, excessive disinfection or repeated disinfection will cause waste of medical resources (Raoofi et al. 2023). The root cause of these problems lies in the lack of data‐supported precise intervention capabilities in traditional nursing. Under the traditional nursing model, data related to IPC are stored separately on different platforms. This fragmented data management makes it difficult for hospitals to accurately identify nodes of cost waste, further pushing up the hidden costs of IPC (He et al. 2022).

With the rapid development of big data, artificial intelligence, and the Internet of Things (IoT) technologies, real‐time data analysis has become one of the core technologies driving transformations in the medical field. By conducting real‐time collection, integration, analysis, and early warning of multi‐dimensional data generated during medical processes, it provides a new solution for precision medicine, risk prevention and control, and cost optimization (Piaggio et al. 2023; Yuan et al. 2024). In the field of intensive care, real‐time data analysis systems can monitor indicators such as patients' heart rate, blood pressure, and blood oxygen saturation, reducing the infection rate in neonatal wards by 7.39% (Tang et al. 2023). In the field of infectious disease prevention and control, real‐time data analysis can integrate case data, transportation data, and environmental data to achieve accurate traceability of epidemics and prediction of transmission trends, providing a scientific basis for the formulation of prevention and control strategies (Said 2023; Ghattas 2025). These successful cases indicate that real‐time data analysis can break through the limitations of traditional medical models and realize the dynamization, precision, and efficiency of medical services through data‐driven approaches.

In the field of ophthalmology, although the application of real‐time data analysis is still in its initial stage, it has shown great potential. The large amount of data generated during ophthalmic diagnosis and treatment provides a rich data source for real‐time data analysis (Lin et al. 2023). From the perspective of nursing practice, this study systematically evaluates the application effect of real‐time data analysis in ophthalmic IPC, providing empirical evidence for the refined and standardized development of ophthalmic IPC nursing. Meanwhile, it explores the feasible path of integrating real‐time data analysis into the practice of ophthalmic IPC nursing and provides operable practical plans for clinical nursing staff. It is expected to provide new ideas and methods for the practice of ophthalmic IPC nursing, promote the transformation of ophthalmic IPC from “experience‐driven” to “data‐driven,” and ultimately achieve the standardized and refined improvement of ophthalmic IPC nursing quality, so as to provide safer, higher‐quality, and more efficient nursing services for patients.

## 2 Materials and Methods

### 2.1 General Information

**FIGURE 1:**
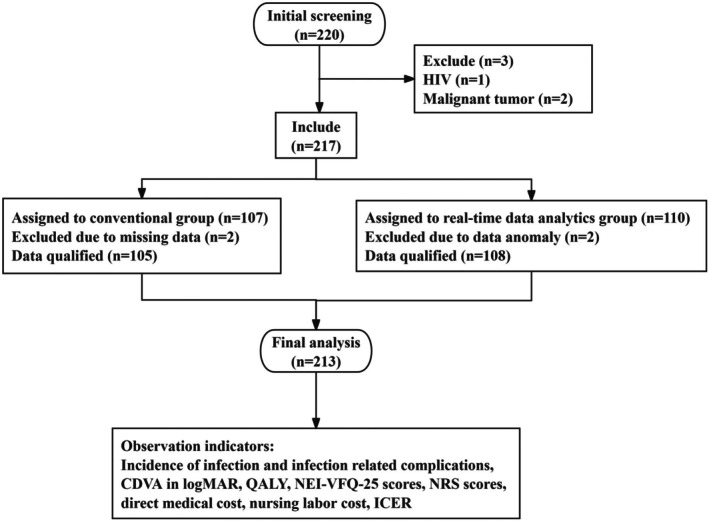
Research flowchart. A total of 220 patients were initially screened in this study, after exclusion, 217 cases were included, among which 2 cases had missing data and 2 cases had data anomaly, ultimately, a total of 213 cases were analyzed, with 105 in conventional group, 108 in real‐time data analytics group. CDVA, corrected distance visual acuity; HIV, human immunodeficiency virus; ICER, incremental cost effectiveness ratio; LogMAR, logarithm of the minimum angle of resolution; NEI‐VFQ‐25, national eye institute visual functioning questionnaire‐25; NRS, numerical rating scale; QALY, quality‐adjusted life year.

This study focused on patients who underwent invasive ophthalmic surgery from January 2022 to December 2024, aiming to investigate the application effect of real‐time data analysis in ophthalmic IPC. Their past medical records were screened, and the study flow chart is shown in Figure 1. A total of 220 patients were initially selected through preliminary screening; after exclusions, 217 patients remained. During the data analysis phase, 2 patients were excluded due to missing medical record data, and another 2 patients were excluded due to abnormal data. Finally, 213 patients were included in the retrospective comparative analysis. According to the different methods of IPC, the patients were divided into two groups: the conventional group (105 patients) received conventional IPC during the nursing period, and the real‐time data analytics group (108 patients) received IPC based on real‐time data analysis during the nursing period.

### 2.2 Inclusion Criteria

(1) Patients who underwent invasive ophthalmic surgery; (2) Aged 18–70 years; (3) Normal mental state and intelligence; (4) No use of systemic antibiotics within 2 weeks before surgery (Kaplan et al. 2022); (5) Patients' expense lists traceable in the hospital financial system; (6) Patients' medical records traceable.

### 2.3 Exclusion Criteria

(1) Patients with immunocompromise, including those complicated with AIDS, malignant tumors (e.g., leukemia, lymphoma), long‐term treatment with immunosuppressants, or long‐term use of glucocorticoids; (2) Patients with severe underlying diseases, including those complicated with severe diabetes, severe cardiovascular and cerebrovascular diseases, chronic renal failure, and decompensated liver cirrhosis; (3) Patients with underlying ocular diseases, including those with pre‐existing ocular infectious diseases (e.g., keratitis, conjunctivitis, endophthalmitis), ocular malignant tumors (e.g., retinoblastoma, conjunctival squamous cell carcinoma), or severe ocular surface diseases (e.g., severe dry eye, limbal stem cell deficiency) before surgery; (4) Patients receiving emergency diagnosis and treatment; (5) Patients who received multiple ophthalmic diagnoses and treatments; (6) Patients with blindness (Sabharwal et al. 2024; Sankarananthan et al. 2023).

### 2.4 Ethical Statement

This study was approved by the Medical Ethics Committee of Shanghai Eye Diseases Prevention & Treatment Center. All patients included in the study signed a written informed consent before the initiation of diagnosis and treatment, agreeing that their clinical data and follow‐up assessment could be used for ethically compliant retrospective research. The study was conducted in strict accordance with the principles of the Declaration of Helsinki. All patient privacy and personal information were anonymized, and there were no ethical or data integrity concerns.

### 2.5 Treatment Methods

This is a single‐center retrospective study. Both groups of patients were recruited from the ophthalmology department of the same hospital. The main reason for receiving different IPC models (manual monitoring model vs. data‐driven model) was the phased implementation of interventions. The conventional group included 105 patients who visited the hospital between January 2022 and June 2023. During this period, the real‐time data analysis system had not yet been introduced in our ophthalmology department, and IPC relied entirely on manual monitoring, empirical assessment, and routine interventions by nursing staff. The real‐time data analytics group included 108 patients who visited the hospital between July 2023 and December 2024. During this period, the real‐time data analysis system was officially launched in the ophthalmology department and fully integrated into IPC nursing practice. Patients in both groups were from the same ophthalmology ward, received diagnosis and care from the same medical and nursing team, and the diagnosis and treatment environment, nursing procedures, staffing, and other conditions remained consistent.

**FIGURE 2:**
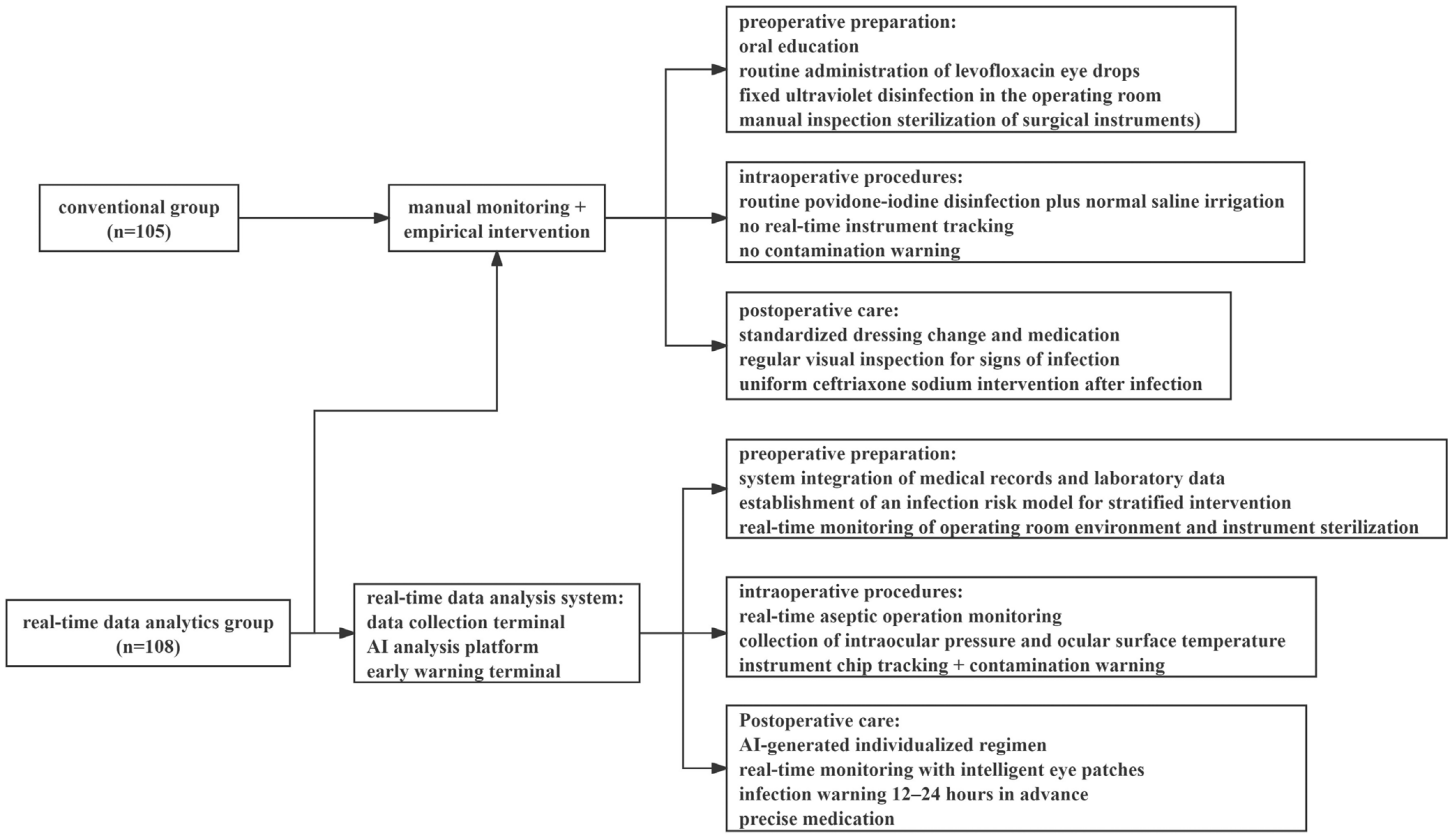
IPC protocols in two groups. The figure illustrates the IPC protocols used in the two groups. The conventional group (105 patients) received conventional IPC during nursing, while the real‐time data analytics group (108 patients) received IPC based on real‐time data analysis. AI, artificial intelligence.

All surgeries were performed by 3 associate chief physicians or higher in the ophthalmology department of our hospital, each with more than 5 years of ophthalmic surgical experience. The three physicians have long been engaged in routine ophthalmic diagnosis, treatment and surgery, with unified surgical protocols and comparable surgical skills. All surgeries were conducted in strict accordance with ophthalmic surgical guidelines. No junior surgeons or surgeons with significant differences in skill level were involved, thus minimizing the impact of inter‐surgeon variability in skill on postoperative infection rates (Figure 2).

During the nursing period, patients in the conventional group adopted the traditional “manual monitoring + empirical intervention” IPC model commonly used in clinical ophthalmology. All infection prevention and control measures were implemented based on the subjective experience of nursing staff and the hospital's basic nursing standards, without real‐time data support or dynamic adjustments. On the basis of the basic nursing procedures of the conventional group, the real‐time data analytics group integrated a real‐time data analysis system (composed of “data collection terminals + AI [Artificial Intelligence] analysis platform + early warning terminals”). The terminals of this system and the intelligent IoT solution were constructed with the assistance of Zhenqu Big Health (Suzhou Zhenqu Technology Information Co. Ltd., China) (Figure 2).

### 2.6 Outcome Measures

#### 2.6.1 Infection Rate and Infection‐Related Complication Rate

Ophthalmic infections are mainly classified into ocular surface infections, intraocular infections, and cavity infections. For ocular surface infections with typical symptoms, a clinical diagnosis can be made directly 3; for those with atypical symptoms, conjunctival sac secretions or corneal scrapings are collected, cultured at 37°C for 24–48 h, and a diagnosis is confirmed if the colony count observed under a microscope is ≥ 10^3^ CFU/mL and pathogenic bacteria are identified (contaminating bacteria are excluded). For intraocular infections, aqueous humor is extracted via anterior chamber paracentesis or vitreous humor via vitrectomy, followed by smear preparation, culture, and drug sensitivity testing; a diagnosis is confirmed if pathogenic bacteria are detected. For cavity infections, secretions from the lacrimal sac area are subjected to smear preparation and culture, and pathogenic bacteria are identified. Severe infection‐related complications among patients who underwent invasive ophthalmic surgery include corneal ulcers, corneal perforation, and symblepharon (Willmann et al. 2025). The number of cases with such complications was counted, and the complication rate was calculated.

#### 2.6.2 Best Corrected Distance Visual Acuity (CDVA)

The measurement of CDVA is conducted in a dedicated visual acuity examination room with an area of no less than 10 m^2^. It is ensured that the distance between the visual acuity chart and the patient's standing position meets the standard (usually 6 m). The internationally standardized logarithm of the minimum angle of resolution (LogMAR) visual acuity chart is adopted, with visual targets being black letters. The size of the visual targets in each row decreases in accordance with logarithmic rules, and the LogMAR values corresponding to adjacent rows of visual targets differ by 0.10. The measurement range generally spans from 0.1 to 2.0, where 0.1 indicates the best visual acuity and 2.0 indicates the worst visual acuity (Hammer et al. 2025).

#### 2.6.3 Quality‐Adjusted Life Year (QALY)

QALY is a life expectancy weighted by quality of life. It is often used in cost‐effectiveness analysis to evaluate health benefits, assist in the rational allocation of medical resources, and maximize benefits with fewer resources. The EuroQol 5‐Dimension 5‐Level (EQ‐5D‐5L) scale is used to measure health status and convert it into a utility index, and then QALY is calculated based on the duration of health status and the utility index of each group (Gutierrez‐Delgado et al. 2021).

#### 2.6.4 National Eye Institute Visual Function Questionnaire‐25 (NEI‐VFQ‐25)

Developed by the National Eye Institute (NEI) of the United States, the NEI‐VFQ‐25 is a specialized quality‐of‐life assessment tool for ophthalmology. It consists of 25 items, covering 11 core domains (including 1 domain for overall health self‐assessment), plus 1 item for visual acuity level assessment. This questionnaire comprehensively evaluates patients' vision‐related quality of life. The total score of the scale can be calculated and converted to a 0–100 scoring system, where a higher score indicates better visual function and fewer discomforts (Goldstein et al. 2022).

#### 2.6.5 Numerical Rating Scale (NRS) Score for Ocular Pain

Ocular pain is one of the core symptoms in patients with infections after ophthalmic surgery. NRS is an internationally used pain quantification tool. Through an intuitive scale of 0–10 points, it accurately reflects the intensity of patients' ocular pain, with higher scores indicating stronger pain intensity (Betz et al. 2023).

#### 2.6.6 Direct Medical Costs, Nursing Labor Costs, and Incremental Cost‐Effectiveness Ratio (ICER)

Direct medical costs refer to the expenses of medical resources directly consumed for preventing surgery‐related infections and treating postoperatively occurring infections (including complications), which are based on the records in patients' expense lists, as well as the costs of the real‐time analytics system (hardware, software, and maintenance). Nursing labor costs refer to the economic costs converted from the time invested by nursing staff in implementing surgical infection prevention and control (preoperative‐intraoperative‐postoperative), monitoring infection conditions, and performing infection nursing operations, calculated according to standardized salaries. The core of ICER is the additional cost required to gain one more unit of QALY, which is calculated based on direct medical costs and QALY (Liu et al. 2023). ICER was calculated as Cost/QALY with non‐parametric bootstrap (1000 replications) for uncertainty.

### 2.7 Sample Size Calculation Method

In a study on the cost‐effectiveness of descemet stripping endothelial keratoplasty (van der Zee et al. 2024), based on the change in QALY (0.80 ± 0.22 vs. 0.90 ± 0.16), the estimated Cohen's *d* was 0.52. The sample size was calculated using the analysis software G*Power 3.1.9.7. With the *α* value set at 0.05, the test power at 90%, and a two‐tailed test, it was calculated that 79 cases were needed in each group, with a total sample size of 158 cases. Considering the potential data loss in retrospective studies, the final analysis data of this study included 105 cases in the conventional group and 108 cases in the real‐time data analytics group, both of which were higher than the theoretical estimates and met the statistical requirements. Moreover, the total sample size (213 cases) was much larger than the theoretical total sample size (158 cases), which could ensure that the results were reliable and stable. During the study, we simultaneously observed several clinical outcomes as secondary endpoints, including infection rate, visual acuity, pain score, and visual function score. We performed a post hoc power analysis during the statistical analysis phase to evaluate the statistical power for these secondary endpoints. The results showed that the statistical power for between‐group comparisons of the key secondary endpoints (infection rate, complication rate, visual acuity, pain score, etc.) was > 80% with the current sample size. This indicates that the sample size provided sufficient statistical power for the secondary endpoints to reliably detect true differences between groups, supporting the stability and reliability of the results.

### 2.8 Statistical Methods

Statistical analysis of patients' baseline data and observation indicators was performed using SPSS 27 software. For measurement data, if they conformed to a normal distribution, they were expressed as mean ± SD. Independent samples *t*‐test was used for comparison between groups, and paired *t*‐test was used for the same group before and after treatment. For measurement data with non‐normal distribution, the Mann–Whitney *U* test was used for comparison between groups, expressed as *M* (IQR). If the ICER value deviated from the normal distribution, the non‐parametric bootstrap method was adopted. For categorical count data, they were presented as *n* (%), and the Chi‐Square test was used for comparison between groups. Fisher's exact test was used for categorical outcomes with expected cell counts < 5. Data were corrected for multiple testing using the Bonferroni method. All data results were considered statistically significant when *p* < 0.05.

## 3 Results

### 3.1 Comparison of Patients' Baseline Data

This study was a retrospective comparative analysis. Baseline information was collected from patients' medical records, including age, Body Mass Index (BMI), gender, type of medical insurance, history of diabetes, and surgical method. All data are presented in Table 1. A comparison of the data between the two groups showed that there were no statistically significant differences in the aforementioned baseline data (all *p* > 0.05). All 95% Confidence Interval (CI) for baseline characteristics crossed the null value (0 for continuous variables, 1 for categorical variables), and combined with *p* > 0.05, indicated that the two groups were balanced and comparable at baseline. The absolute effect sizes of all baseline variables were close to 0 and extremely small, suggesting negligible practical differences between the two groups in demographics, comorbidities, and surgical types. This further confirmed balanced group allocation and provided a reliable baseline for subsequent comparisons of intervention effects.

**TABLE 1:** Baseline data [mean ± SD, *n* (%)].

Variables	Conventional group (*n* = 105)	Real‐time data analytics group (*n* = 108)	95% CI	*p*	Effect size
Age (years)	47.31 ± 7.49	47.16 ± 8.28	−1.98, 2.29	0.885	0.020
BMI (kg/m^2^)	23.71 ± 1.67	23.65 ± 1.68	−0.39, 0.51	0.789	0.037
Gender					
Male	55 (52.4)	57 (52.8)	0.58, 1.69	0.954	−0.004
Female	50 (47.6)	51 (47.2)			
Insurance type					
Resident medical insurance	56 (53.3)	58 (53.7)	0.58, 1.69	0.957	−0.004
Employee medical insurance	49 (46.7)	50 (46.3)			
Diabetes	25 (23.8)	28 (25.9)	0.48, 1.66	0.721	−0.024
Surgical type					
Cataract phacoemulsification surgery	53 (50.5)	55 (50.9)	—	0.599	0.094
Vitreous retinal surgery	27 (25.7)	25 (23.1)			
Glaucoma trabeculectomy	24 (22.9)	24 (22.2)			
Keratoplasty	1 (1.0)	4 (3.7)			

Abbreviation: BMI, body mass index.

### 3.2 Multivariate Regression Analysis Adjusted for Confounding Factors

Table 2 presents the results of a multivariate logistic regression analysis on the association between the real‐time data analytics‐based infection prevention and control (IPC) model and postoperative infection after adjusting for confounding factors. Postoperative infection was set as a binary outcome variable (infection = 1, no infection = 0). Variables included in the model were age, BMI, gender, type of medical insurance, diabetes mellitus, type of surgery, intervention measure, and surgeon grade. The results showed that after simultaneous adjustment for the above confounding factors, the infection risk in the real‐time data analytics group was significantly lower than that in the conventional group (OR = 0.42, 95% CI: 0.24–0.73, *p* = 0.002). Among other covariates, age, BMI, gender, type of medical insurance, diabetes mellitus, type of surgery, and surgeon grade showed no statistically significant association with postoperative infection (all *p* > 0.05). The *p*‐value of the Hosmer–Lemeshow goodness‐of‐fit test was 0.847 (> 0.05), indicating a satisfactory model fit without significant deviation. These findings confirm that the real‐time data analytics‐driven IPC model exerts an independent and significant protective effect against reducing ophthalmic postoperative infection, even after adjusting for potential confounding factors such as surgeon experience.

**TABLE 2:** Multivariate regression analysis adjusted for confounding factors.

Variables	*β*	OR (95% CI)	*p*
Age	0.371	1.45 (0.89, 2.36)	0.136
BMI	0.322	1.38 (0.91, 2.09)	0.128
Gender	0.140	1.15 (0.74, 1.78)	0.532
Insurance type	−0.084	0.92 (0.59, 1.43)	0.712
Diabetes	−0.128	0.88 (0.55, 1.25)	0.142
Surgical type	−0.117	0.89 (0.41, 1.33)	0.325
Treatments	−0.868	0.42 (0.24, 0.73)	0.002
Surgeon grade	−0.401	0.67 (0.32, 1.42)	0.526
Hosmer‐Lemeshow goodness‐of‐fit test *p*‐value	0.847		

### 3.3 Infection Rate and Infection‐Related Complication Rate in the Two Groups

Based on patients' medical records, as shown in Table 3, in the conventional group, postoperative infections included 4 cases of ocular surface infection, 3 cases of intraocular infection, and 2 cases of cavity infection, with a total of 9 infection cases and an infection rate of 8.6%. In the real‐time data analytics group, postoperative infections included 1 case of ocular surface infection and 1 case of intraocular infection, with a total of 2 infection cases and an infection rate of 1.9%. A comparison between the two groups revealed that the infection rate of the real‐time data analytics group was significantly lower than that of the conventional group (95% CI: 1.12, 21.45, *p* = 0.032). Among patients with infections, the number of severe infection‐related complications was counted. In the conventional group, there were 2 cases of corneal ulcers, 1 case of corneal perforation, and 1 case of symblepharon, totaling 4 cases; no severe infection‐related complications occurred in the real‐time data analytics group. The incidence of infection‐related complications in the real‐time data analytics group was reduced than that in the conventional group but *p* = 0.057 after appropriate testing (95% CI: 0.89, 2.85). In the present results, the 95% CIs for both the total infection rate and the total incidence of infection‐related complications did not cross 1, and *p* < 0.05, indicating that the between‐group differences in infection and complication control were statistically significant in favor of the real‐time data analytics group over the conventional group. The effect sizes were 0.142 and 0.132, respectively, representing small‐to‐moderate effect sizes, which suggests that real‐time data analysis has clinical value in reducing ophthalmic infections and related complications. The above results indicate that the application of real‐time data analysis can reduce the occurrence of infections during ophthalmic surgical treatment.

**TABLE 3:** Comparison of infection incidence rate and incidence of infection complications [*n* (%)].

Variables		Conventional group (*n* = 105)	Real‐time data analytics group (*n* = 108)	95% CI (exact method)	Fisher's exact test (*p*)	Effect size	Bonferroni correction (*p*)
Infection	Eye surface infection	4 (3.8)	1 (0.9)	0.28, 15.82	0.212	—	−0.636
	Intraocular infection	3 (2.9)	1 (0.9)	0.21, 26.45	0.354	—	1.000
	Genital infection	2 (1.9)	0 (0.0)	0.41, ∞	0.242	—	0.726
	Total	9 (8.6)	2 (1.9)	1.12, 21.45	0.032	0.142	0.032
Infection complications	Corneal ulcer	2 (1.9)	0 (0.0)	0.41, ∞	0.242	—	0.726
	Corneal perforation	1 (0.9)	0 (0.0)	0.06, ∞	0.500	—	1.000
	Eyelid adhesion	1 (0.9)	0 (0.0)	0.06, ∞	0.500	—	1.000
	Total	4 (3.8)	0 (0.0)	0.89, 2.85	0.057	0.132	0.114

### 3.4 Comparison of CDVA in LogMAR Between the Two Groups

The CDVA in LogMAR of the two groups was compared, and the results are shown in Table 4. There was no significant difference in CDVA in LogMAR between the two groups before surgery (95% CI: −0.06, 0.05, *p* = 0.804). One month after surgery, the CDVA in LogMAR of both groups decreased significantly (all *p* < 0.05). Furthermore, it can be observed that the decrease in the real‐time data analytics group (0.15 ± 0.07) was more significant than that in the conventional group (0.17 ± 0.08) (95% CI: 0.00, 0.04, *p* = 0.042). The 95% CI for preoperative CDVA in LogMAR crossed 0, with *p* > 0.05 and an effect size close to 0, indicating that the two groups were balanced and comparable in baseline visual acuity. At 1 month postoperatively, the 95% CI for CDVA in LogMAR did not cross 0 and *p* < 0.05, indicating a statistically significant between‐group difference. The effect size was 0.280 (small‐to‐moderate effect size), suggesting that real‐time data analytics–based IPC has a positive impact on postoperative visual recovery. The above results suggest that the application of real‐time data analysis can help patients undergoing ophthalmic surgery recover their visual acuity more quickly.

**TABLE 4:** Comparison of CDVA in LogMAR (mean ± SD).

Variables	Time	Conventional group (*n* = 105)	Real‐time data analytics group (*n* = 108)	95% CI	*p*	Effect size	Bonferroni correction (*p*)
CDVA in LogMAR	Before surgery	0.40 ± 0.19	0.40 ± 0.20	−0.06, 0.05	0.804	−0.034	0.804
	1 month after surgery	0.17 ± 0.08*	0.15 ± 0.07*	0.00, 0.04	0.042	0.280	0.042

Abbreviations: CDVA, corrected distance visual acuity; LogMAR, logarithm of the minimum angle of resolution.

* *p* < 0.05 vs. before surgery.

### 3.5 Comparison of QALY Between the Two Groups

The QALY results are shown in Table 5. Before surgery, there was no difference in QALY between the two groups (95% CI: −0.04, 0.04, *p* = 0.887). One month after surgery, the health status of patients in both groups improved significantly (all *p* < 0.05), and the increase in the real‐time data analytics group (0.84 ± 0.13) was more significant than that in the conventional group (0.80 ± 0.14) (95% CI: −0.08, −0.003, *p* = 0.036). The 95% CI for preoperative QALY crossed 0, with *p* > 0.05 and an effect size close to 0, indicating that the two groups were balanced in baseline quality of life before surgery. At 1 month postoperatively, the 95% CI for QALY did not cross 0 and *p* < 0.05, suggesting a statistically significant between‐group difference. The effect size was −0.290 (small‐to‐moderate effect size), indicating that real‐time data analytics intervention was associated with improved postoperative QALYs in patients. These results suggest that the application of real‐time data analysis can better improve the health status and quality of life of patients undergoing ophthalmic surgery.

**TABLE 5:** Comparison of QALY (mean ± SD).

Variables	Time	Conventional group (*n* = 105)	Real‐time data analytics group (*n* = 108)	95% CI	*p*	Effect size	Bonferroni correction (*p*)
QALY	Before surgery	0.70 ± 0.14	0.70 ± 0.14	−0.04, 0.04	0.887	0.020	0.887
	1 month after surgery	0.80 ± 0.14*	0.84 ± 0.13*	−0.08, −0.003	0.036	−0.290	0.036

Abbreviation: QALY, quality‐adjusted life year.

* *p* < 0.05 vs. before surgery.

### 3.6 Comparison of NEI‐VFQ‐25 Scores Between the Two Groups

The statistical analysis of the NEI‐VFQ‐25 score data evaluated in patients' medical records is presented in Table 6. Before surgery, there was no significant difference in NEI‐VFQ‐25 scores between the two groups (95% CI: −0.84, 0.80, *p* = 0.956). One month after surgery, the NEI‐VFQ‐25 scores of patients in both groups increased significantly (all *p* < 0.05). A between‐group comparison of the two groups one month after surgery showed that the NEI‐VFQ‐25 score of the real‐time data analytics group (77.21 ± 4.02 scores) was significantly higher than that of the conventional group (76.10 ± 3.52 scores) (95% CI: −2.14, −0.10, *p* = 0.032). The 95% CI for preoperative NEI‐VFQ‐25 crossed 0, with *p* > 0.05 and an effect size close to 0, indicating that the two groups were balanced and comparable in baseline visual function–related quality of life before surgery. At 1 month postoperatively, the 95% CI for NEI‐VFQ‐25 did not cross 0 and *p* < 0.05, indicating a statistically significant between‐group difference. The effect size was −0.296 (small‐to‐moderate effect size), suggesting that real‐time data analytics intervention was associated with improved postoperative visual function–related quality of life in patients.

**TABLE 6:** Comparison of NEI‐VFQ‐25 scores (mean ± SD, scores).

Variables	Time	Conventional group (*n* = 105)	Real‐time data analytics group (*n* = 108)	95% CI	*P*	Effect size	Bonferroni correction (*p*)
NEI‐VFQ‐25	Before surgery	69.85 ± 2.97	69.87 ± 3.09	−0.84, 0.80	0.956	−0.008	0.956
	1 month after surgery	76.10 ± 3.52*	77.21 ± 4.02*	−2.14, −0.10	0.032	−0.296	0.032

Abbreviation: NEI‐VFQ‐25, national eye institute visual functioning questionnaire‐25.

* *p* < 0.05 vs. before surgery.

### 3.7 Comparison of NRS Scores Between the Two Groups

The NRS scores of the two groups were compared, and the results are shown in Table 7. There was no significant difference in NRS scores between the two groups before surgery (95% CI: −0.36, 0.30, *p* = 0.866). One month after surgery, the NRS scores of both groups decreased significantly (all *p* < 0.05). Furthermore, it can be observed that compared with the conventional group (1.49 ± 0.96 scores), the decrease in the real‐time data analytics group (1.19 ± 0.91 scores) was more significant (95% CI: 0.04, 0.54, *p* = 0.024). For preoperative NRS pain scores, the 95% CI crossed 0, *p* > 0.05, and the effect size was close to 0, indicating that the two groups were balanced and comparable in baseline pain severity before surgery. For 1‐month postoperative NRS scores, the 95% CI did not cross 0 and *p* < 0.05, indicating a statistically significant between‐group difference. The effect size was 0.040, suggesting that real‐time data analytics–based IPC was associated with reduced postoperative ocular pain in patients. These results suggest that the pain of patients in both groups was improved after surgery, and the application of real‐time data analysis resulted in a better improvement effect.

**TABLE 7:** Comparison of NRS scores (mean ± SD, scores).

Variables	Time	Conventional group (*n* = 105)	Real‐time data analytics group (*n* = 108)	95% CI	*p*	Effect size	Bonferroni correction (*p*)
NRS scores	Before surgery	2.98 ± 1.22	3.01 ± 1.23	−0.36, 0.30	0.866	−0.023	0.866
	1 month after surgery	1.49 ± 0.96*	1.19 ± 0.91*	0.04, 0.54	0.024	0.040	0.040

Abbreviation: NRS, numerical rating scale.

* *p* < 0.05 vs. before surgery.

### 3.8 Comparison of Direct Medical Costs and Nursing Labor Costs Between the Two Groups

The results of the analysis on patients' direct medical costs and nursing labor costs are shown in Table 8. The direct medical cost and nursing labor cost of patients in the conventional group were 3246.35 ± 157.69 yuan and 606.02 ± 192.57 yuan, respectively; the direct medical cost and nursing labor cost of patients in the real‐time data analytics group were 2197.29 ± 167.16 yuan and 358.07 ± 192.14 yuan, respectively. A comparison between the two groups revealed that the reduction in both costs was more significant in the real‐time data analytics group (for direct medical costs: 95% CI: 1005.14, 1092.97, *p* < 0.001; for nursing labor costs: 95% CI: 195.99, 299.92, *p* < 0.001). The 95% CIs for both direct medical costs and nursing labor costs did not cross 0, and *p* < 0.001, indicating highly statistically significant differences between the real‐time data analytics group and the conventional group. The effect sizes were 6.453 and 1.289, respectively, both representing large effect sizes, suggesting that real‐time data analytics was associated with reductions in medical costs and nursing labor costs. It can be concluded that IPC using real‐time data analysis can reduce the direct medical costs and nursing labor costs of patients undergoing ophthalmic surgery.

**TABLE 8:** Comparison of direct medical cost and nursing labor cost (mean ± SD, yuan).

Variables	Conventional group (*n* = 105)	Real‐time data analytics group (*n* = 108)	95% CI	*p*	Effect size	Bonferroni correction (*p*)
Direct medical cost	3246.35 ± 157.69	2197.29 ± 167.16	1005.14, 1092.97	< 0.001	6.453	< 0.001
Nursing labor cost	606.02 ± 192.57	358.07 ± 192.14	195.99, 299.92	< 0.001	1.289	< 0.001

### 3.9 Comparison of ICER Between the Two Groups

The median direct medical cost in the real‐time data analysis group was 2197.29 yuan (IQR: 1950.80–2480.60), which was lower than 3246.35 yuan (IQR: 2890.50–3680.20) in the conventional group, with an incremental cost of −1049.06 yuan (95% bootstrap CI: −1385.42 to −712.70). Meanwhile, the median QALY in the real‐time data analysis group was 0.61 (IQR: 0.54–0.68), higher than 0.52 (IQR: 0.45–0.58) in the conventional group, with an incremental effectiveness of 0.09 QALY (95% CI: 0.04–0.14). Since the real‐time data analysis group achieved both lower costs (saving approximately 1049 yuan) and better effectiveness (gaining an additional 0.09 QALY), the incremental cost‐effectiveness ratio (ICER) was −11656.22 yuan/QALY, indicating a dominant economic result. This demonstrates that the intervention holds an absolute cost‐effectiveness advantage (Table 9).

**TABLE 9:** Comparison of ICER [*M* (IQR), yuan/QALY].

Variables	Conventional group (*n* = 105)	Real‐time data analytics group (*n* = 108)	Incremental difference	ICER	95% bootstrap CI
Direct medical cost (yuan)	3246.35 (2890.50, 3680.20)	2197.29 (1950.80, 2480.60)	−1049.06		(−1385.42, −712.70)
QALY	0.52 (0.45, 0.58)	0.61 (0.54, 0.68)	0.09		(0.04, 0.14)
ICER				−11656.22	Dominant

Abbreviation: ICER, incremental cost effectiveness ratio.

## 4 Discussion

The results of this study suggested that the real‐time data analytics group exhibited significantly lower infection and infection‐related complication rates than the conventional group, validating the core value of this model in IPC. Unlike traditional nursing—where infection risk assessment relies on subjective postoperative signs (e.g., ocular redness, discharge), leading to delayed intervention due to atypical early infection symptoms—the real‐time analytics model enables proactive risk mitigation. Its multidimensional monitoring system (integrating patient baseline data, surgical process metrics, and postoperative dynamics) captures subtle risk signals and triggers automated alerts, prompting prioritized risk investigation before the onset of overt symptoms (Gheorghe 2024). This targeted intervention aligns with Thandar et al.'s (2022) findings (RR = 0.65, 95% CI: 0.45–1.07), reinforcing the necessity of data‐driven strategies to optimize IPC interventions.

Beyond reducing infections, real‐time analytics directly advanced the ultimate goals of ophthalmic nursing: preserving visual function and enhancing quality of life. The analytics group demonstrated superior LogMAR CDVA and NEI‐VFQ‐25 scores, alongside lower ocular pain NRS scores. By mitigating infection risk at the source, this model minimized inflammatory disruptions to visual recovery (e.g., corneal edema, refractive abnormalities) and prevented severe (Baudouin et al. 2021). It further improved patient‐centered outcomes by dynamically adjusting care plans—optimizing analgesic dosing and dressing change frequency—based on real‐time visual recovery trends and pain scores, thereby reducing functional limitations and boosting postoperative comfort (Ting et al. 2023).

Amid constrained healthcare resources, the model also demonstrated strong cost‐effectiveness: the real‐time analytics group had lower direct medical and nursing labor costs per infected patient, with a more favorable ICER. Early warning and timely intervention avoided unnecessary resource utilization (e.g., additional antibiotics, intravitreal injections, prolonged hospitalization) (Felfeli et al. 2023), while automated data collection/analysis streamlined workflows—freeing nurses from manual data tasks to focus on patient education and personalized care. The conventional group's higher infection rate raised direct medical costs, while the real‐time data analytics group reduced these costs by preventing infections and their progression via early warning. Traditional IPC nursing involves time‐consuming, error‐prone manual data work; the real‐time system enables “automatic data collection + real‐time analysis + early warning prompts,” freeing nurses for patient‐centered care and cutting labor costs. ICER is core for evaluating medical intervention cost‐effectiveness (Kruzikas et al. 2020). The superior ICER indicates that the health gains (reduced infections, improved visual prognosis) far outweigh the incremental investment, consistent with El Khatib et al. (2023) conclusion that intelligent analysis models correlate significantly with improved medical performance (*t* = 4.31, *p* < 0.05). Based on China's per capita GDP of approximately 89 358 yuan in 2024, the 1–3 times willingness‐to‐pay (WTP) threshold ranged from 89 000 to 268 000 yuan per QALY gained. The incremental cost‐effectiveness ratio (ICER) in this study was −11656.22 yuan/QALY, which was far below the one‐time GDP threshold and negative, indicating that the real‐time data analysis group was dominant and economically superior at any reasonable WTP threshold.

## 5 Study Limitations

Although this study provides empirical reference for the data‐driven transformation of ophthalmic IPC nursing, it still has multi‐dimensional limitations from the perspectives of clinical research methodology standards, conclusion reliability, and practical promotion. These limitations not only affect the generalizability of the study conclusions but also point out the direction for improvement in subsequent studies. The detailed analysis can be carried out from the following three aspects:

1. This study only selected patients from our centre as the research subjects, resulting in a lack of population representativeness in the sample. There are significant institutional differences in the hardware configuration and nursing level of ophthalmic diagnosis and treatment. The demographic characteristics of patients in a single center are prone to centralized bias, and retrospective studies themselves have selection bias. Furthermore, no control was implemented for confounding variables in this study, so a causal relationship for the results cannot be established. If the conclusions obtained on this basis are directly promoted to primary hospitals, their actual effects may be greatly reduced.
2. This study only conducted a 1‐month postoperative follow‐up, which belongs to short‐term efficacy evaluation and cannot cover the long‐term impact chain of ophthalmic infections. The lack of long‐term health outcomes makes it impossible to determine whether the data system can reduce the risk of long‐term complications. Additionally, the study only calculated short‐term costs and did not include long‐term investments in the data system, such as system upgrade fees, nursing staff data training fees, and equipment maintenance fees, leading to doubts about the cost‐effectiveness of the ICER conclusions.
3. The study did not consider the resource constraints of primary hospitals, resulting in the inability to apply the conclusions to primary‐level medical institutions. The initial investment in the data system far exceeds the budget capacity of primary hospitals; moreover, primary hospitals lack data maintenance personnel, which increases operational costs.

Future studies should be improved in the following aspects: conduct multi‐center, prospective controlled trials, balance confounding factors such as doctor operation skills and patient compliance through random allocation, extend the follow‐up period to 1–2 years, and include indicators of long‐term visual prognosis and drug resistance rate; develop differentiated monitoring protocols for different ophthalmic diseases to enhance the reproducibility of the research; carry out adaptability studies in primary hospitals, design low‐cost simplified data systems, conduct subgroup analyses, and clarify application strategies for different populations. Only through the above improvements can the value of real‐time data analysis be verified more objectively and comprehensively, promoting its transition from single‐center pilot to large‐scale clinical application, and truly providing reliable support for the refined development of ophthalmic IPC nursing.

In terms of nursing workload, traditional IPC relies on manual documentation, manual verification, and empirical rounds by nursing staff, which is not only cumbersome and time‐consuming but also prone to increasing infection risks due to human error. In contrast, the real‐time data analytics system can automatically collect and integrate multi‐dimensional perioperative monitoring data with real‐time alerts, greatly reducing the time nurses spend on repetitive data management and repeated checks (Valek et al. 2023). While lowering nursing labor costs, this allows nurses to focus more on high‐value clinical nursing tasks such as patient education, pain management, and complication monitoring, effectively optimizing the allocation of nursing resources. Regarding clinical decision‐making, traditional nursing mostly depends on subjective experience to assess infection risk, which is vulnerable to atypical early symptoms and individual judgment differences, leading to delayed intervention. Real‐time data analytics can dynamically monitor key information including patients' comorbidities, surgical procedures, and postoperative signs, accurately identify high‐risk nodes, and provide quantitative evidence to assist nurses in making objective, timely, and targeted intervention decisions (Yuan et al. 2024). For nursing standardization, real‐time data analytics embeds key IPC nodes, operational standards, and alert thresholds into the monitoring process, unifying the assessment, intervention, and evaluation criteria for perioperative infection prevention and control. It reduces discrepancies in implementation among different nurses and shifts, promoting the transformation of ophthalmic IPC nursing from a decentralized, individualized experience‐based model to a standardized, homogenized, and refined modern nursing model, providing a solid practical foundation for establishing a sustainable and replicable ophthalmic infection prevention and control nursing system (Ghattas 2025).

## 6 Conclusion

Real‐time data analysis provides a new technical approach for ophthalmic IPC nursing. Its value lies not only in reducing the infection rate, but more importantly in promoting the transformation of the nursing model—from passive response to active prevention and control, and from experience‐based decision‐making to data‐driven decision‐making. In the future, more high‐quality research and practical exploration are needed to further unleash the potential of data technology and provide safer, more efficient, and more economical nursing services for ophthalmic patients.

## 7 Relevance for Clinical Practice

From a nursing perspective, this model provides nurses with quantitative metrics and intervention points, reducing subjective judgment errors and facilitating the promotion of unified nursing standards. For hospital management, it offers a basis for resource allocation. Prioritizing its application in high‐risk departments may help alleviate resource constraints. For patients, it reduces infection‐induced visual impairment at the source, enhances patient trust and satisfaction with treatment, and provides a reference for improving the quality of ophthalmic care.

## Author Contributions

**Zhenhui Chen:** conceptualization. **Shengcheng Wu:** conceptualization, methodology. **Yi Zhou:** software, formal analysis. **Chun Tang:** writing – original draft, writing – review and editing.

## Funding

The authors have nothing to report.

## Disclosure

No generative or artificial intelligence tools were used in the preparation, writing, editing, translation, data analysis, or figure/table creation of this manuscript.

## Ethics Statement

The study has been reviewed and approved by the Ethics Committee of Shanghai Eye Diseases Prevention & Treatment Center (Approval number: 2024SQ023).

## Consent

Informed consent has been waived with the approval of the Ethics Committee.

## Conflicts of Interest

The authors declare no conflicts of interest.

## Data Availability

The data supporting the findings of this study can be obtained from the corresponding author, upon request.
